# Identification of pleiotropic and specific therapeutic targets for cardio-cerebral diseases: A large-scale proteome-wide mendelian randomization and colocalization study

**DOI:** 10.1371/journal.pone.0300500

**Published:** 2024-05-31

**Authors:** Yanchen Zhu, Yahui Wang, Zhaorui Cui, Fani Liu, Jiqiang Hu

**Affiliations:** Cardiology Department, Dongfang Hospital, Beijing University of Chinese Medicine, Beijing, China; Brigham and Women’s Hospital and Harvard Medical School, UNITED STATES

## Abstract

**Background:**

The cardiac-brain connection has been identified as the basis for multiple cardio-cerebral diseases. However, effective therapeutic targets for these diseases are still limited. Therefore, this study aimed to identify pleiotropic and specific therapeutic targets for cardio-cerebral diseases using Mendelian randomization (MR) and colocalization analyses.

**Methods:**

This study included two large protein quantitative trait loci studies with over 4,000 plasma proteins were included in the discovery and replication cohorts, respectively. We initially used MR to estimate the associations between protein and 20 cardio-cerebral diseases. Subsequently, Colocalization analysis was employed to enhance the credibility of the results. Protein target prioritization was based solely on including highly robust significant results from both the discovery and replication phases. Lastly, the Drug-Gene Interaction Database was utilized to investigate protein-gene-drug interactions further.

**Results:**

A total of 46 target proteins for cardio-cerebral diseases were identified as robust in the discovery and replication phases by MR, comprising 7 pleiotropic therapeutic proteins and 39 specific target proteins. Followed by colocalization analysis and prioritization of evidence grades for target protein, 6 of these protein-disease pairs have achieved the highly recommended level. For instance, the PILRA protein presents a pleiotropic effect on sick sinus syndrome and Alzheimer’s disease, whereas GRN exerts specific effects on the latter. APOL3, LRP4, and F11, on the other hand, have specific effects on cardiomyopathy and ischemic stroke, respectively.

**Conclusions:**

This study successfully identified important therapeutic targets for cardio-cerebral diseases, which benefits the development of preventive or therapeutic drugs.

## Introduction

Cardio-cerebral diseases considerably burden human health, with therapeutic challenges remaining daunting. The count of cardiovascular diseases (CVDs) cases increased from 271 million in 1990 to 523 million in 2019 globally [1]. Besides CVDs, cerebral disorders represent another field that impacts human health, resulting in disability and leading causes of morbidity [2,3]. A plethora of literature indicates that disturbances in the cardiovascular system negatively impact brain function [4–6]. The cardiac-brain connection, or heart-brain axis, has been identified as the basis for multiple cardio-cerebral diseases with genetic associations across domains [7,8]. However, effective drug targets and therapeutic approaches for most cardio-cerebral diseases remain severely limited due to multifactorial etiology, sudden or comorbid onset, and elusive pathophysiology.

The measurability and quantifiability of plasma proteins in the circulating blood endow them with potential regulatory agents or therapeutic targets for cardio-cerebral diseases [9]. However, randomized controlled trials have been slow to elucidate the causal effects of large-scale plasma proteins on multiple disease etiologies. The implementation of the method poses challenges due to extended trial periods, great expense, and ethics. Furthermore, confounding biases during the study, such as educational background and socioeconomic status, significantly impact the risk of cardio-cerebral diseases and cannot be quantified and adjusted [10–12]. Additionally, many cardio-cerebral diseases can change plasma protein levels via individual behaviors and biological pathways, which may interfere with observed associations and lead to reverse causal relationships. Hence, a more dependable and replicable research methodology is required to elucidate the plasma protein function in treating diverse cardio-cerebral ailments.

Genetics-driven genomics studies can accelerate drug development with advancements in genomics. Mendelian randomization (MR) analyses, reducing bias from confounding and reverse causation, are used to study disease-relevant molecular mediators [13]. Recent large-scale population studies targeting genetic variants associated with plasma protein levels, known as protein quantitative trait loci (pQTL), have provided an opportunity to identify target proteins causally related to disease outcomes [9,14]. Researchers can use MR analyses to elucidate better causal relationships between circulating proteins and diseases, as well as identify drug targets and therapeutic aims for managing cardio-cerebral.

In this study, we leveraged two large-scale pQTL datasets (Ferkingstad, E. et al. and Zhang et al.) as well as the latest extensive genome-wide association study (GWAS) data for 20 common clinical cardio-cerebral diseases [9,14]. Our study proposes identifying a spectrum of pleiotropic and specific prioritized therapeutic proteins for cardio-cerebral diseases utilizing MR and colocalization analyses. This provides robust evidence to identify potential pharmacological targets and therapeutic modalities to navigate the complex overlap between cardio-cerebral disease states and their co-morbidities.

## Methods

### Study design and ethics

**Fig 1:**
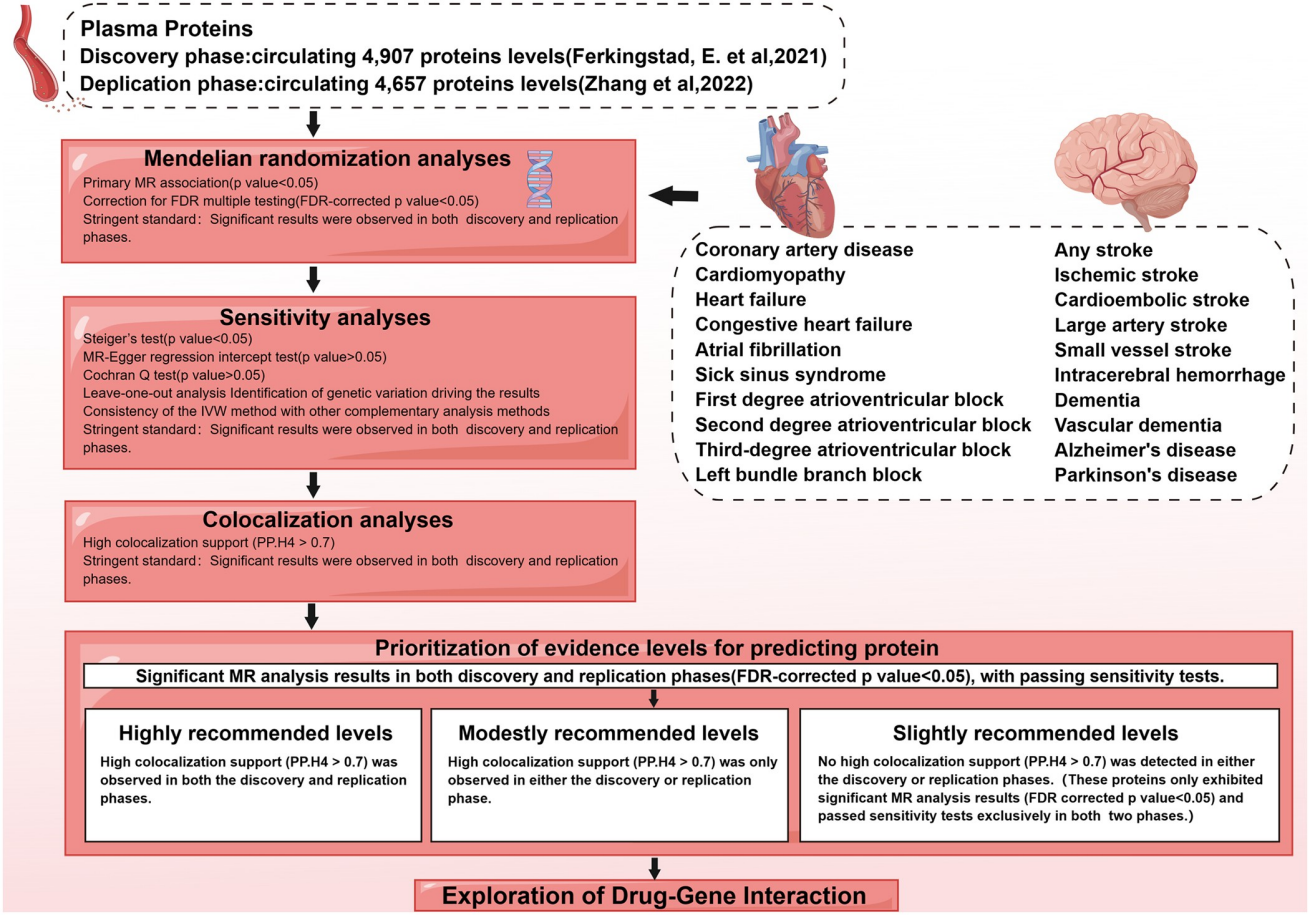
Overview of the study’s design (by Figdraw). FDR, false discovery rate; PP.H4: Posterior probability for hypotheses 4 (based on Bayesian model).

Fig 1 illustrates the study design. The studies included in the analysis were approved by their respective ethical review committees. Given that this study analyzed publicly available summary data that did not use raw data, so the ethical approval is deemed unnecessary.

### Plasma protein quantitative trait loci data source

The present investigation utilized summary-level statistics used a plasma large-scale pQTL analysis conducted by Ferkingstad, E. et al. in 2021, which involved a cohort of 35,559 individuals of Icelandic descent [9]. A total of 4,907 plasma proteins were measured using the SomaScan multiplex aptamer assay (version 4). Variants associated with the germline genome sequences were excluded to account for the unique inheritance patterns of the sex chromosomes. During the replication phase, the cis-heritability summary statistics of 4,657 plasma proteins were incorporated into a cohort of 7,213 European American (EA) individuals sourced from the multi-ethnic dataset provided by Zhang et al. in 2022 [14]. This investigation was based on plasma protein levels from 7,213 European American (EA) and 1,871 African American individuals from the Atherosclerosis Risk in Communities (ARIC) study. It exclusively focused on protein-coding genes of autosomal loci with minor allele frequency (MAF) exceeding 1% within a cis-regulatory region defined as the transcription start site ± 500 kb.

### Diseases genome-wide association study data source

In this study, a systematic collection of the most recent and comprehensive 20 GWAS summary data on cardio-cerebral diseases, specifically European ancestry, was conducted. The total sample size ranged from 267,018 to 1,308,460. These diseases include coronary artery disease, cardiomyopathy, heart failure, congestive heart failure, atrial fibrillation, sick sinus syndrome, first-degree atrioventricular block, second-degree atrioventricular block, third-degree atrioventricular block, left bundle branch block, any stroke, ischemic Stroke, cardioembolic stroke, large artery stroke, small vessel stroke, intracerebral hemorrhage, dementia, vascular dementia, Alzheimer’s disease, and Parkinson’s disease [15–20]. All outcome data were binary variables, including the effect allele frequency (EAF). Further elaboration on all outcome GWAS data is presented in S1 Table.

### Selection of genetic variants and variants harmonization

The present study utilized the plasma pQTL dataset of Ferkingstad et al. to identify potential instrumental variables. This was achieved by conducting a genome-wide association study within a 500 kb window upstream and downstream of the protein-coding chromosome starting location (GRCh38) for single nucleotide polymorphisms (SNPs) that exhibited significant association with exposure (P < 1×10^−05^) and had a MAF over 1%. To eliminate any potential confounding effects, SNPs that exhibited a strong association with the outcome (P<5×10^−06^) were subsequently removed. The European LD reference panel was employed for the 1000 Genomes Project Phase 3 using the ieugwasr R package (version 0.1.5). The objective was to ensure that the instrumental variables were genetically independent, using a 1000 kb window and paired LD r2 < 0.1. Subsequently, the R package Mendelian Randomization (version 0.7.0) was employed to conduct a genetic variant complementation analysis. This involved utilizing the beta values of the same effect allele of the protein tool variables and the corresponding outcome while removing ambiguous palindromic SNPs. Finally, the variance of the tool variables was quantified using the r2, and the strength of the tool variables was quantified using the F-statistic [21]. An F-statistic greater than 10 indicated the absence of weak bias in the instrumental variables. The same criteria were employed during the replication phase to select genetic variants and coordinate genetic variation, utilizing Zhang et al.’s plasma pQTL datasets.

### Primary MR analyses

MR analyses were utilized as the primary method to infer causal relationships between exposure and outcome in our study’s discovery and replication cohorts. The STROBE-MR Statement was consulted to guide the implementation of Primary MR Analysis for each exposure-outcome correlation [22,23]. The number of available instrumental variables determined the selection of modeling strategies. Specifically, the Wald ratio model was employed for exposure-outcome pairs featuring at least one instrumental variable, while the fixed inverse-variance weighted (Fix-IVW) model was utilized for pairs with two to three instrumental variables. The random IVW model was employed for pairs with four or more instrumental variables. Furthermore, MR Egger, Weighted Median, and Weighted Mode models were employed as directional validation for the primary IVW analysis for pairs with multiple instrumental variables [24–26]. Odds ratios (OR) and 95% confidence intervals (CI) were converted into effect sizes and standard errors due to the binary nature of all outcomes examined in this study. Subsequently, multiple testing was conducted adjustment utilizing the false discovery rate (FDR) method with a 5% threshold to mitigate the possibility of false positive discoveries. The significant findings were defined as those with an FDR-corrected P value below 0.05.

### Sensitivity analyses

Initially, the Steiger test was utilized to mitigate the potential impact of reverse causality on our MR findings [27]. This statistical method was used to evaluate the contribution of instrumental variables to the variance of the exposure and outcome, thereby ascertaining the presence or absence of reverse causality. A P value of less than 0.05 from the MR Steiger test indicated that the results are not susceptible to reverse causation. Additionally, a data filtration process was implemented to exclude evidence variance that exhibited a stronger association with the outcome than with the exposure, thereby enhancing the reliability of our MR analyses. The MR-Egger regression intercept test was employed as an additional measure during a protein exposure group analysis involving multiple instrumental variables [24]. If the P value of the test result exceeded 0.05, it indicated a lack of statistical significance between the intercept and zero, implying multiple effects. Consequently, these results were excluded from consideration. Subsequently, heterogeneity tests (Cochran Q test) revealed the presence of some heterogeneity in the outcomes. However, eliminating any results from using the random-IVW model was unnecessary for MR analysis with multiple instrumental variables. Ultimately, leave-one-out analysis was employed to verify a solitary instrumental variable that did not influence the findings, and the outcomes were reexamined after eliminating the influential factor.

Following the execution of sensitivity analyses and FDR correction on the outcomes of our primary MR analysis, the durability of these MR findings was substantiated. MR outcomes were exclusively acknowledged that have undergone concurrent FDR correction and sensitivity analysis as dependable. The Mendelian Randomization R package (version 0.7.0) was employed to conduct all aforementioned analyses.

### Colocalization analyses

Colocalization analyses were conducted using the coloc R package (version 5.2.1) to augment the level of evidence for the significant results [28]. The Bayesian model was employed to ascertain the linkage disequilibrium-driven association between the identified proteins and diseases. The protein loci were defined as the cis-regulatory regions encoding the protein genes. The colocalization Bayesian model operates on the assumption that a maximum of one causal SNP is linked to each trait in the locus region. Consequently, five mutually exclusive hypotheses were developed: Hypothesis (H0), positing the absence of any SNP associated with both the protein and disease; H1, suggesting the presence of an SNP associated solely with the protein; H2, proposing the existence of an SNP associated solely with the disease; H3, postulating the presence of distinct SNPs associated with the protein and disease; and H4, positing the presence of a single SNP associated with both the protein and disease [29]. The prior probabilities were set to 1×10^−04^ for p1 when a SNP was related only to trait one, 1×10^−04^ for p2 when a SNP was solely related to trait two, and 1×10^−05^ for p12 when a SNP was related to both traits. If the posterior probability for H4 (PP.H4) exceeded 0.7, the SNP was considered to have strong colocalization evidence with both traits.

### Prioritization of evidence levels for predicting protein

The proteins were detected using MR analysis and integrated using FDR result correction and sensitivity analysis into our protein target repository to facilitate a dependable evaluation of protein targets. The identified proteins were categorized into three distinct and mutually exclusive tiers of substantiation. Initially, proteins identified as noteworthy in the discovery and replication analyses, as well as MR and colocalization analyses, were classified as highly recommended level protein targets. Subsequently, proteins deemed significant in MR analysis during the discovery and replication phases but only in one of the colocalization analysis phases were classified as modestly recommended level protein targets. Proteins that were significant in MR analysis in the discovery and replication groups but did not pass the colocalization analysis were eventually classified as slightly recommended level protein targets.

### Exploration of a drug-gene interaction

Protein gene information was systematically searched and compared at various levels utilizing the Drug-Gene Interaction Database (DGIdb) (https://www.dgidb.org/) to comprehensively investigate the potential value of the identified proteins in drug therapy. This database amalgamates data from various sources, such as expert-curated information, text mining, drug-gene interactions, and gene function information, and assigns priority to potential therapeutic genes [30]. This methodology facilitates a comprehensive comprehension of the potential application of the identified proteins (or genes) in drug therapy and supports further research in this domain.

## Results

### MR and sensitivity analyses estimated the potential causal effects of plasma protein on cardio-cerebral diseases

Data on pQTLs for 1,931 proteins with accessible instrumental variables were extracted from Ferkingstad et al.’s dataset of 4,907 plasma proteins during the discovery phase. Subsequently, we conducted meticulous MR analyses between 1,931 proteins and 20 cardio-cerebral disease-related outcomes. Our analyses used a stringent F-statistic threshold of at least 10 and an FDR-corrected P value of less than 0.05. A total of 144 proteins have significant associations with the outcomes in the primary MR analysis following the implementation of Steiger tests to eliminate the potential for reverse causality. Subsequently, MR-Egger regression intercept tests were conducted for proteins with multiple instrumental variables as exposure data, resulting in the exclusion of 20 results that were susceptible to pleiotropy. Additionally, heterogeneity tests (Cochran Q tests) revealed that 81% of the significant results lacked heterogeneity. According to a leave-one-out analysis, the outcomes of our research were not significantly influenced by instrumental variables, thus establishing the reliability of our conclusions. Additionally, we have reinforced the accuracy of our findings utilizing comparison models, specifically MR Egger, Weighted Median, and Weighted Mode models. Ultimately, our sensitivity analyses have confirmed the validation of 124 proteins, and we have deemed these results robust because they have successfully passed the false discovery rate correction and sensitivity analysis.

During the replication phase, we adhered rigorously to established benchmarks and subjected the protein data provided by Zhang et al. to meticulous analysis. Following the filtration of available instrumental variables, we successfully extracted 1,805 pQTL datasets from 4,657 proteins, which successfully replicated 1750 (91%) available pQTL datasets at the discovery stage. Our subsequent MR analysis aimed to explore potential associations between these proteins and cardio-cerebral diseases, resulting in 128 notable findings. We can detect and eliminate 19 potentially pleiotropic outcomes using additional sensitivity analysis. Heterogeneity testing did not reveal any significant deviations from our anticipated outcomes. Furthermore, we have strengthened the precision of our findings by comparing the outcomes of other models. Our analysis has produced 109 reliable MR outcomes that merit further investigation.

**Fig 2:**
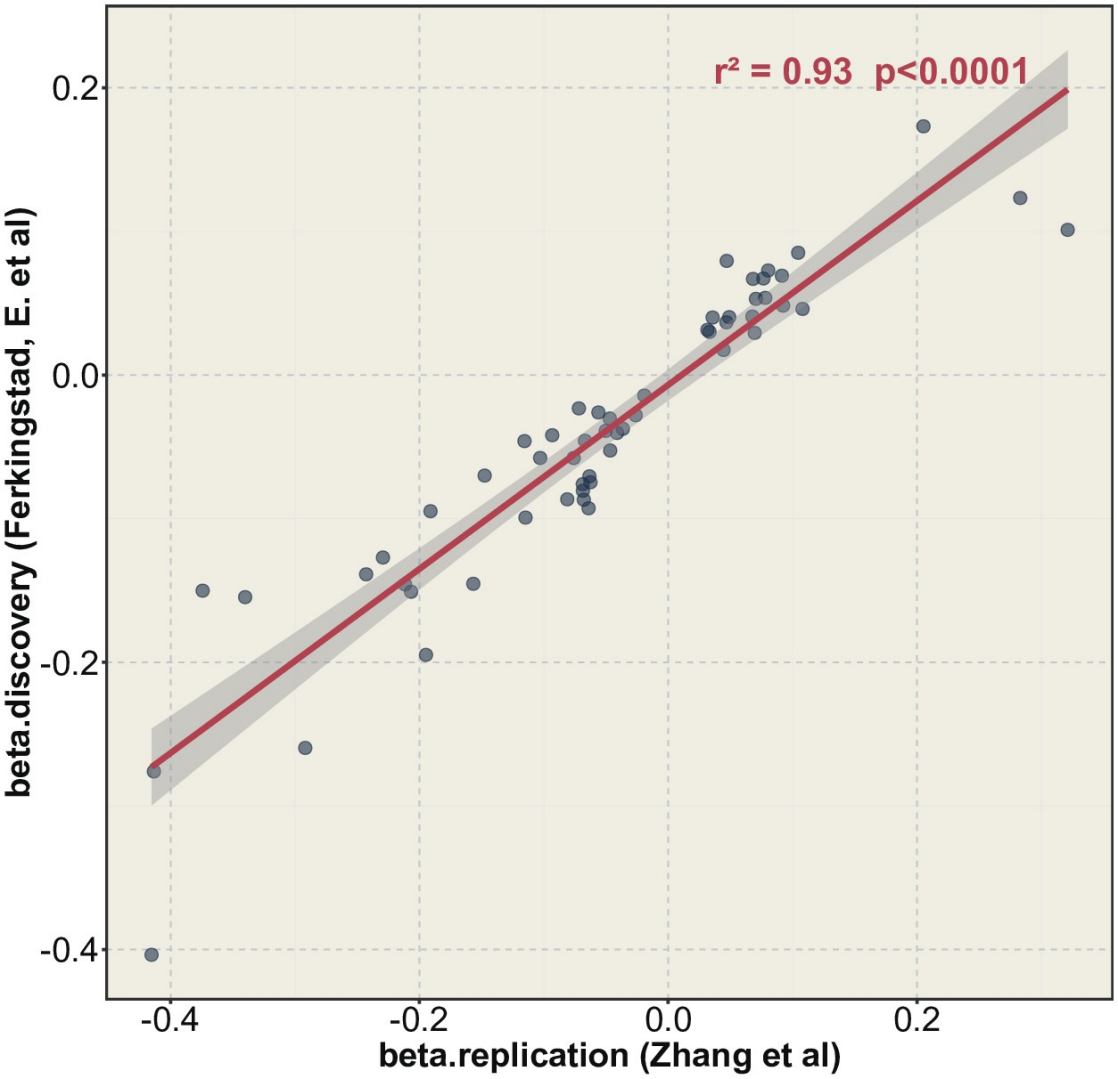
Scatter plots of the correlations between beta values of protein-disease effects during the discovery and replication phases. The protein-disease effect values exhibited a strong concordance for all results across the discovery and replication phases. The correlation calculated was highly significant using Pearson’s method, with an r^2^ value of 0.93 and a P value of 1.52×10^−25^.

In this investigation, we have identified noteworthy MR results that exhibit resilience across the discovery and replication phases. Our analysis encompassed 54 protein-disease associations exhibiting considerable significance in both phases. Afterward, we conducted a correlation examination utilizing the two-phase outcomes’ effect sizes (beta) and generated a scatter plot. The correlation coefficient and P value was computed using the Pearson method, yielding remarkably consistent MR estimates that attest to the dependability of both Ferkingstad et al.’s discovery analysis and Zhang et al.’s replication analysis (Fig 2). The protein targets identified encompassed 23 for coronary heart disease, one for sick sinus syndrome, eight for atrial fibrillation, two for heart failure subtypes, 12 for stroke subtypes, and wight for dementia subtypes.

### Advanced colocalization analyses validated highly recommended protein targets

We utilized colocalization algorithms for additional scrutiny. We employed identical prior probabilities and significance thresholds in both analysis phases to augment the evidentiary support for protein-disease associations derived from our discovery and replication analyses. The aforementioned prior probabilities and significance thresholds were consistently applied throughout the analytical process. The results of the co-localization analyses supported 12 of the 54 protein-disease pairs previously identified. Notably, six pairs presented significant corroborative evidence favoring the H4 hypothesis during the discovery and replication phases. These pairs encompassed one pair for cardiomyopathy, two for ischemic stroke, one for sick sinus syndrome, and two for Alzheimer’s disease.

### Prioritization of highly recommended level for predicting protein and exploration of a drug-gene interaction

The study’s protein target library has incorporated 54 protein-disease pairs that have exhibited robustness in replication and discovery analyses. The prioritization of the protein evidence level identified in the database was based on the colocalization analysis results. The corresponding target protein of the six protein-disease pairs with significant co-localization analysis in replication and discovery phases has been identified as highly recommended level protein targets. When colocalization analysis yielded significant outcomes in the discovery or replication phases for six protein-disease pairs, the corresponding target protein was classified as modestly recommended level protein target. Conversely, the remaining 42 protein-disease pairs, exhibiting only robust MR results, identified the corresponding target protein as slightly recommended level protein targets. S2 Table provides additional information concerning protein targets for each level.

### Drug-gene interaction

We conducted a methodical exploration of the DrugGene Interaction Database to identify potential drug-protein target matches based on their prioritized levels because the therapeutic efficacy of most drugs depends on their ability to interact with specific proteins selectively. We aimed to evaluate the feasibility of utilizing the genes as therapeutic targets. Our efforts successfully identified drug information of 16 protein targets. S3 Table illustrates further elaboration.

### Pleiotropic effect of PILRA protein in sick sinus syndrome and Alzheimer’s disease

**Fig 3:**
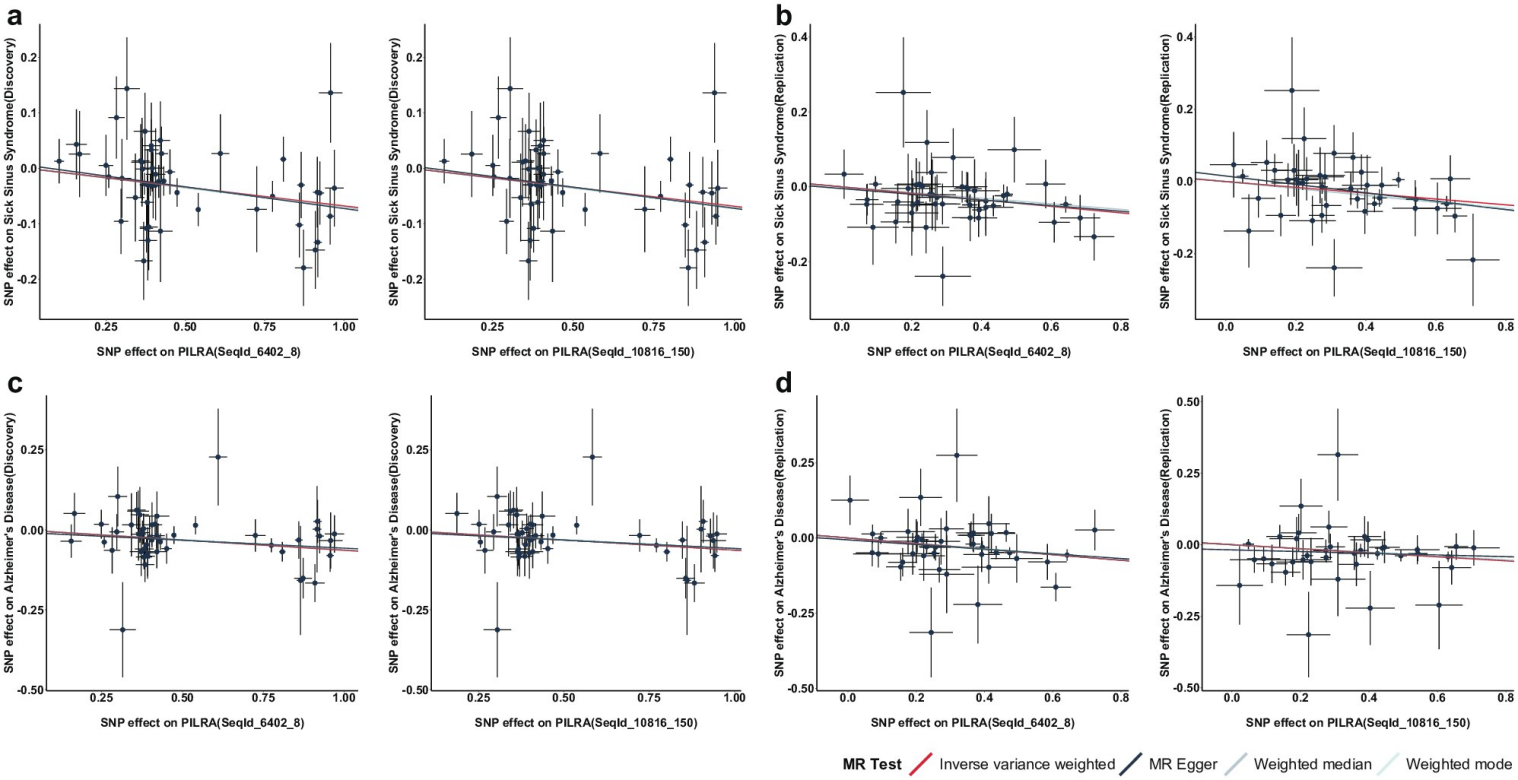
Scatter plots of MR associations of PILRA protein with risk of sick sinus syndrome and Alzheimer’s disease. Two types of Sequence Identifiers (SeqId) were used to map the PILRA protein in this study. SNP, single nucleotide polymorphism.

**Fig 4:**
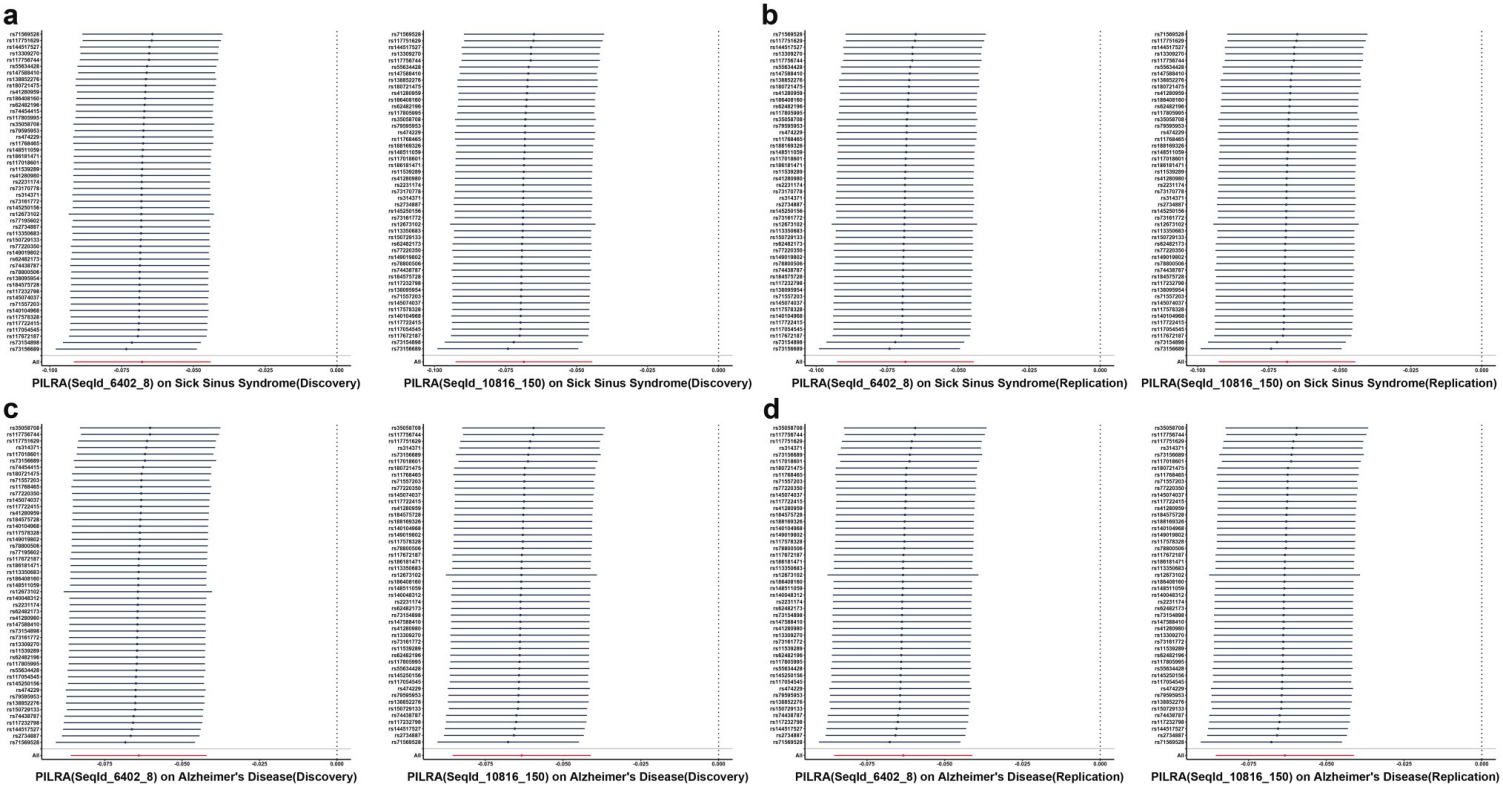
Leave-one-out analyses for PILRA protein on sick sinus syndrome and Alzheimer’s disease. Two types of Sequence Identifiers (SeqId) were used to map the PILRA protein in this study. The dark dots denote the effect measures derived from the IVW-MR analysis, excluding certain SNPs. However, the red lines represent the pooled analysis encompassing all SNPs conducted using the IVW-MR method.

Our study unveiled a strong causal association between the PILRA protein and the development of Sick Sinus Syndrome and Alzheimer’s disease. The F-statistics surpass 10 at both phases, indicating the absence of instrumental variable bias. Further sensitivity analyses have demonstrated that the MR results remain robust by reverse causation bias at either phase and display no evidence of heterogeneity or pleiotropy (S4 Table). According to a leave-one-out analysis, our study has confirmed that no single instrumental variables influence our results, thus establishing the reliability of our findings (Fig 4). Additionally, results maintained consistent outcomes across different models, enhancing our conclusions’ accuracy (Fig 3). The aforementioned outcomes have been substantiated using colocalization investigations (S4 Table), providing highly recommended level of evidence for the robust pleiotropic protein targets of PILRA protein in the context of Sick Sinus Syndrome and Alzheimer’s disease.

### Other proteins with specific effects in highly recommended level

Our study has identified five specific effect protein targets at evidence level one. Notably, our research has established a causal linkage between the APOL3 protein and cardiomyopathy, while the F11 and LRP4 proteins have been identified as potential culprits for ischemic stroke. Additionally, we have observed a robust association between the GRN protein and Alzheimer’s disease, validating the discovery and replication phases of MR analysis, with colocalization evidence supporting both phases (S1 and S2 Figs, S4 Table). The Supplemental table illustrates a more comprehensive understanding of proteins with varying levels of evidence.

## Discussion

Cardio-cerebral diseases pose a significant therapeutic challenge and affect a considerable portion of the global population [1–3]. This study successfully identified a total of 46 prospective target proteins for cardio-cerebral diseases in the discovery and replication phases by MR, comprising 7 pleiotropic therapeutic proteins and 39 specific target proteins. Followed by colocalization analysis and prioritization of evidence grades for predicting protein, 6 of these protein-disease pairs have achieved the highly recommended level. Specifically, the PILRA protein presents a pleiotropic effect on both sick sinus syndrome and Alzheimer’s disease, whereas GRN exerts specific effects on the latter. APOL3, LRP4, and F11, on the other hand, have specific effects on cardiomyopathy and ischemic stroke, respectively, all classified as highly recommended level protein targets.

The present study has identified PILRA protein as a pleiotropic protein target in sick sinus syndrome and Alzheimer’s disease. PILRA, a paired immunoglobulin-like receptor (PIR) family member, functions as an inhibitory receptor and is primarily expressed in immune cells such as B cells, T cells, and natural killer (NK) cells. Its roles encompass immune regulation and inflammation [31]. Prior research has linked PILRA to herpes simplex and inflammatory bowel disease [32,33]. Sick sinus syndrome is a complex cardiac arrhythmia that represents a significant indication for the worldwide adoption of permanent pacemakers [34]. Nevertheless, the etiology and pharmacological treatment of sick sinus syndrome remain subjects of ongoing investigation. In this study, we report for the first time that PILRA protein may also serve as a therapeutic target for sick sinus syndrome and provide highly recommended level evidence. Additionally, a recent study demonstrated that a common missense variant (G78R, rs1859788) of PILRA reduces the binding of PILRA to various ligands, including HSV-1 glycoprotein B, thereby decreasing the risk of HSV-1 infection, which is considered an environmental protect factor for Alzheimer’s Disease [35]. The PILRA R78G-A allele has negatively modified the effect of APOEε4 and GM17 high-risk variants on Alzheimer’s disease risk. APOEε4 and GM17 are genetic risk factors for Alzheimer’s disease and may also affect host susceptibility to herpes simplex virus 1 (HSV-1) infections [36]. Hence, the PILRA gene variant (G78R, rs1859788) holds the potential as a crucial protective locus against the onset of Alzheimer’s disease. Our study corroborates prior research, underscoring the PILRA protein’s efficacy as a robust therapeutic target for Alzheimer’s disease. Our colocalization analyses during the replication and discovery stages of sick sinus syndrome and Alzheimer’s disease confirm that the causal SNP, rs1859788, drives the observed effects. Thus, identifying PILRA as a promising therapeutic target for sick sinus syndrome and Alzheimer’s disease carries significant clinical implications. However, the absence of pharmacological agents targeting PILRA poses a significant challenge in advancing novel treatment strategies. Therefore, there is an urgent need to accelerate the development of PILRA-targeted drugs to address the unmet medical needs of patients afflicted with these debilitating disorders.

The clinical practice of cardio-cerebral diseases reveals their heterogeneity, as the same disease can manifest with varying etiologies, symptoms, severity, and prognosis across individuals. The priority target proteins identified in this study are expected to exhibit efficacy solely in a subset of patients due to their critical involvement in the pathogenesis or progression of the disease. Additionally, certain drug targets may prove ineffective or deleterious in specific populations. Hence, forthcoming investigations pertaining to protein targets must necessitate more advanced scrutiny and assessment. To recapitulate, the prioritized plasma proteins ascertained in this inquiry may indicate auspicious drug targets or function as prognostic biomarkers, thereby furnishing drug targets and therapeutic objectives for the prospective management of intricate cardiovascular and cerebrovascular ailments and multimorbidity.

This study possessed numerous strengths, with a significant advantage being the utilization of MR and colocalization analyses to estimate the causal effects of plasma proteins on multiple complex cardio-cerebral diseases. The MR design, based on randomly allocated and fixed alleles before birth, effectively minimized biases stemming from confounding and reverse causation, thereby enhancing causal inference. Furthermore, rigorous sensitivity analysis and multiple testing corrections were employed to substantiate further the MR hypotheses and the robustness of the findings. Colocalization analysis was also employed to examine the potential influence of linkage disequilibrium on the protein-disease associations, thereby augmenting the robustness of our MR analysis findings. Additionally, the study cohort was limited to individuals of European descent, mitigating any potential confounding effects of population stratification. Furthermore, our research employed two extensive proteomic investigations for discovery and replication analyses. We exclusively chose the most resilient outcomes that displayed high significance in both sets of significant findings, as determined by intersectional and correlation analyses. This approach ensured our research’s dependability and significant reference value for forthcoming clinical studies.

Notably, our study also has some limitations. First, the scope of the investigation was confined to individuals of European descent, thereby constraining the applicability of our discoveries to other ethnic groups. Second, we exclusively utilized cis-regulatory regions as instrumental variables, which may mitigate horizontal pleiotropy to some extent and result in reduced statistical efficacy. Third, while the replication phase of this study effectively validated 91% of the pQTL datasets obtained from the discovery phase, a minor fraction of the pQTL datasets was not retained. Lastly, our inquiry was restricted to identifying and validating pQTL dataset utilizing accessible instrumental variables, potentially neglecting alternative therapeutic targets.

## Conclusions

This study successfully identified 46 prospective target proteins for cardio-cerebral diseases, comprising 7 pleiotropic target proteins and 39 specific target proteins. The study revealed pleiotropic protein targets that may impact multiple disease risks, including sick sinus syndrome, Alzheimer’s disease, and ischemic stroke, as well as high-priority specific proteins that target individual diseases. The present findings offer potential pharmacological targets and therapeutic objectives for the integrated management of intricate cardio-cerebral disorders and comorbidities.

## Supporting information

S1 Fig Scatter plots of MR associations of other protein in the highly recommended level targets with risk of cardio-cerebral diseases. SNP, single nucleotide polymorphism. SeqId, sequence identifiers.
(TIF)

S2 Fig Leave-one-out analyses for other proteins in the highly recommended level targets on cardio-cerebral diseases. The dark dots denote the effect measures derived from the IVW-MR analysis, excluding certain SNPs. The red lines represent the pooled analysis encompassing all SNPs conducted using the IVW-MR method. SeqId, sequence identifiers. IVW, inverse-variance weighted. MR, mendelian randomization.
(TIF)

S1 Table Summary information of diseases genome-wide association study data source. (XLSX)

S2 Table The prioritization of evidence levels for predicting protein and establishing associations between targets and diseases. OR, odds ratio; 95% CI, 95% confidence interval.
(XLSX)

S3 Table Detailed information on drug-gene interaction. (XLSX)

S4 Table Comprehensive data regarding the results of all significant MR analyses in the discovery and replication stages. (XLSX)
