# Supplementary figures and images for: Identification of pleiotropic and specific therapeutic targets for cardio-cerebral diseases: A large-scale proteome-wide mendelian randomization and colocalization study
Source: PLoS One. 2024 May 31;19(5):e0300500. doi: 10.1371/journal.pone.0300500 (PMC11142593; doi:10.1371/journal.pone.0300500)

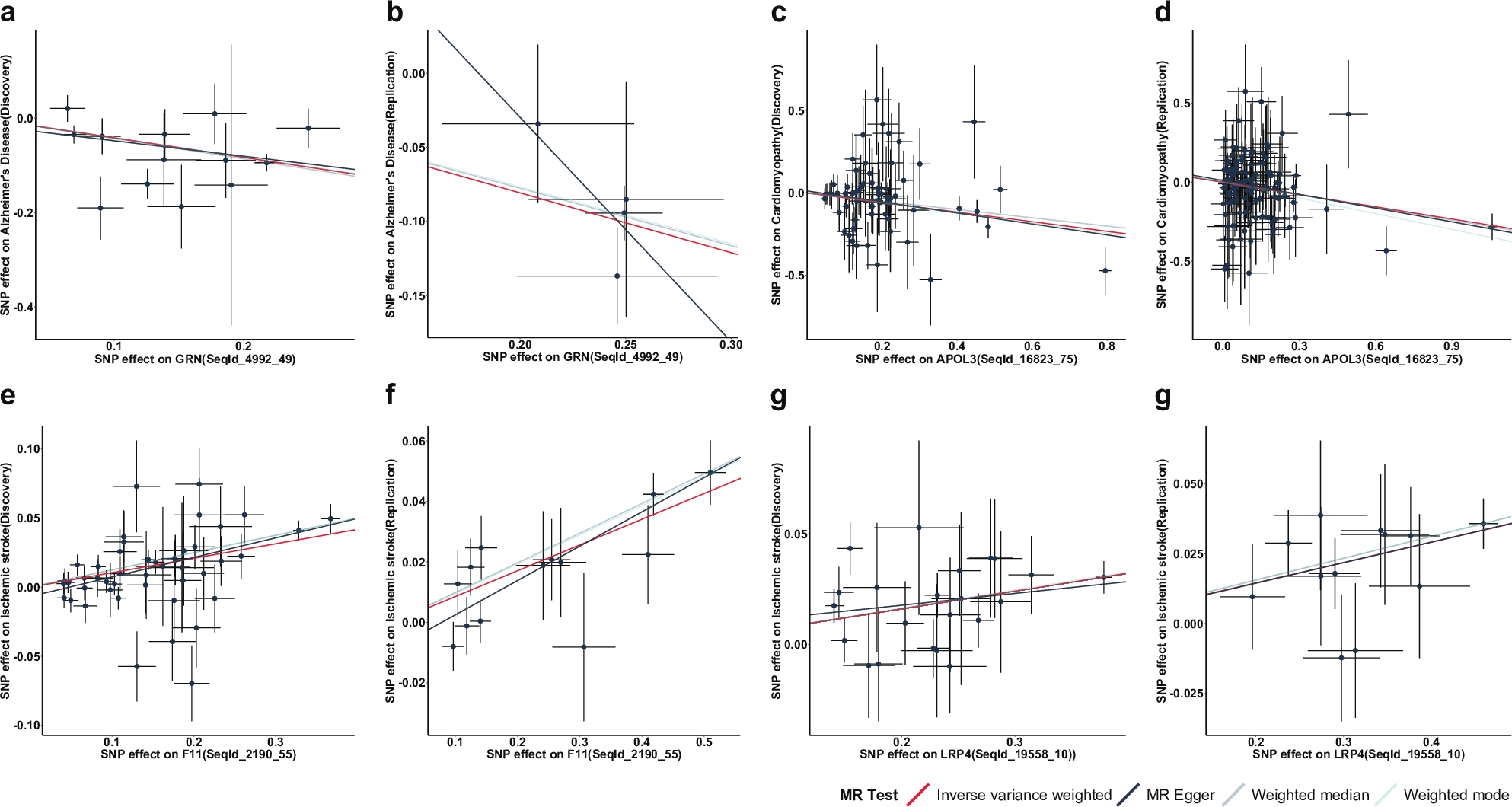

Supplement: S1 Fig — SNP, single nucleotide polymorphism. SeqId, sequence identifiers. (TIF) [file pone.0300500.s001.tif]

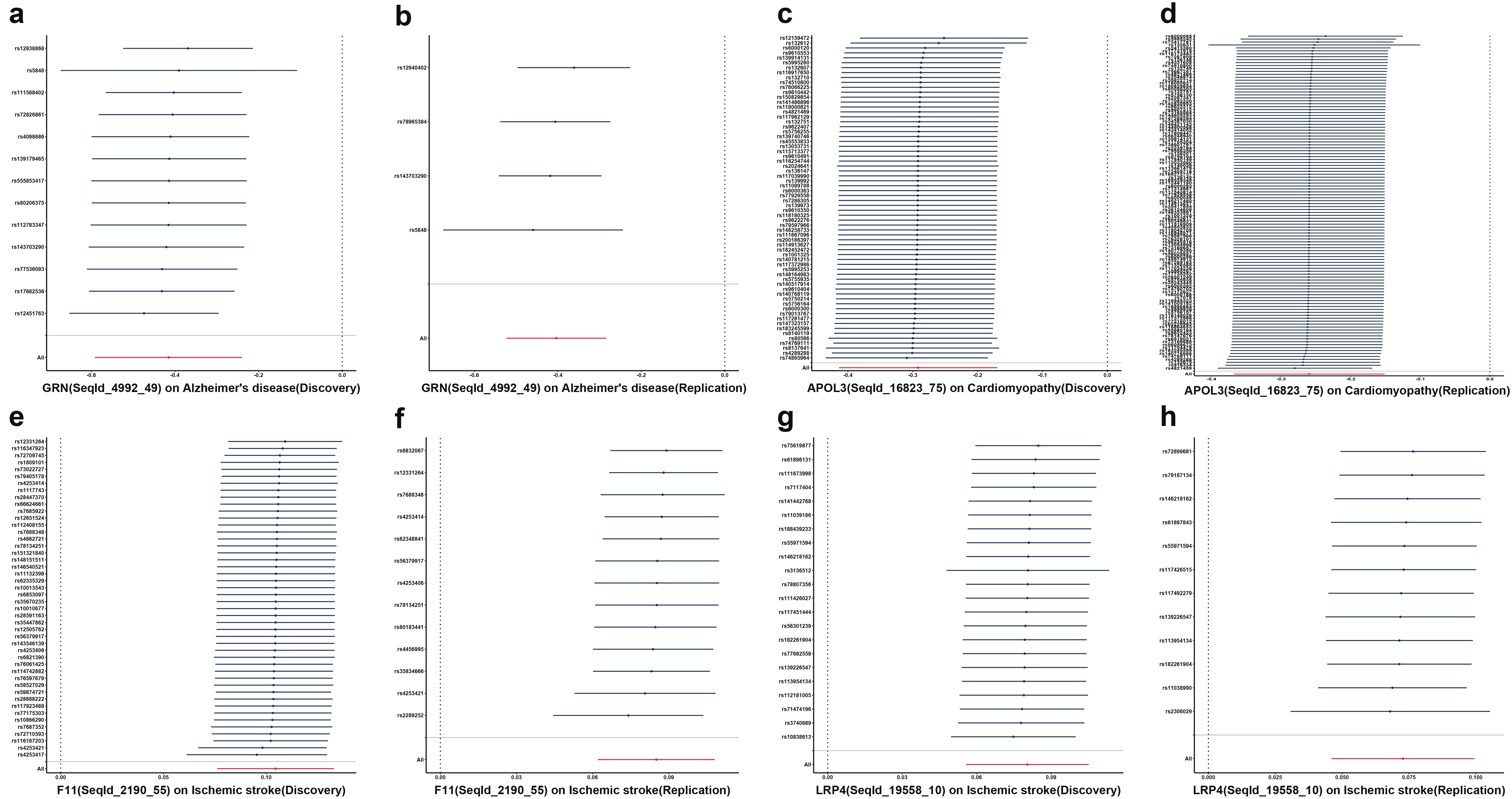

Supplement: S2 Fig — The dark dots denote the effect measures derived from the IVW-MR analysis, excluding certain SNPs. The red lines represent the pooled analysis encompassing all SNPs conducted using the IVW-MR method. SeqId, sequence identifiers. IVW, inverse-variance weighted. MR, mendelian randomization. (TIF) [file pone.0300500.s002.tif]
